# Comparative Evaluation of Tumor-Infiltrating Lymphocytes in Companion Animals: Immuno-Oncology as a Relevant Translational Model for Cancer Therapy

**DOI:** 10.3390/cancers14205008

**Published:** 2022-10-13

**Authors:** Christopher J. Pinard, Andrew Lagree, Fang-I Lu, Jonathan Klein, Michelle L. Oblak, Roberto Salgado, Juan Carlos Pinto Cardenas, Barbara Brunetti, Luisa Vera Muscatello, Giuseppe Sarli, Maria Pia Foschini, Alexandros Hardas, Simon P. Castillo, Khalid AbdulJabbar, Yinyin Yuan, David A. Moore, William T. Tran

**Affiliations:** 1 Department of Clinical Studies, Ontario Veterinary College, University of Guelph, Guelph, ON N1G 2W1, Canada; 2 Odette Cancer Program, Sunnybrook Health Sciences Centre, Toronto, ON M4N 3M5, Canada; 3 Radiogenomics Laboratory, Sunnybrook Health Sciences Centre, Toronto, ON M4N 3M5, Canada; 4 Department of Laboratory Medicine and Pathobiology, University of Toronto, Toronto, ON M5S 1A8, Canada; 5 Department of Radiation Oncology, Albert Einstein College of Medicine and Montefiore Medical Center, Bronx, NY 10461, USA; 6 Division of Research, Peter MacCallum Cancer Centre, Melbourne 3000, Australia; 7 Department of Pathology, GZA-ZNA Hospitals, 2610 Antwerp, Belgium; 8 Department of Pathology, DIAGSA, Mexico City 53910, Mexico; 9 Department of Veterinary Medical Sciences, University of Bologna, 40064 Ozzano dell’Emilia, Italy; 10 Department of Biomedical and Neuromotor Sciences, University of Bologna, 40127 Bologna, Italy; 11 Department of Pathobiology & Population Sciences, The Royal Veterinary College, Hertfordshire AL9 7TA, UK; 12 Centre for Evolution and Cancer, The Institute of Cancer Research, London SM2 5NG, UK; 13 Division of Molecular Pathology, The Institute of Cancer Research, London SM2 5NG, UK; 14 Department of Pathology, UCL Cancer Institute, London WC1E 6DD, UK; 15 University College Hospitals NHS Trust, London NW1 2PG, UK; 16 Department of Radiation Oncology, University of Toronto, Toronto, ON M5T 1P5, Canada; 17 Department of Radiation Oncology, Sunnybrook Health Sciences Centre, Toronto, ON M4N 3M5, Canada; 18 Temerty Centre for AI Research and Education in Medicine, University of Toronto, Toronto, ON M5S 1A8, Canada

**Keywords:** comparative oncology, canine, neoplasia, TILs

## Abstract

**Simple Summary:**

Laboratory experiments studying solid tumors are limited by the inability to adequately model the tumor microenvironment and important immune interactions. Immune cells that infiltrate the tumor bed or periphery have been documented as reliable biomarkers in human studies. Veterinary oncology provides a naturally occurring cancer model that could complement biomarker discovery, clinical trials, and drug development.

**Abstract:**

Despite the important role of preclinical experiments to characterize tumor biology and molecular pathways, there are ongoing challenges to model the tumor microenvironment, specifically the dynamic interactions between tumor cells and immune infiltrates. Comprehensive models of host-tumor immune interactions will enhance the development of emerging treatment strategies, such as immunotherapies. Although in vitro and murine models are important for the early modelling of cancer and treatment-response mechanisms, comparative research studies involving veterinary oncology may bridge the translational pathway to human studies. The natural progression of several malignancies in animals exhibits similar pathogenesis to human cancers, and previous studies have shown a relevant and evaluable immune system. Veterinary oncologists working alongside oncologists and cancer researchers have the potential to advance discovery. Understanding the host-tumor-immune interactions can accelerate drug and biomarker discovery in a clinically relevant setting. This review presents discoveries in comparative immuno-oncology and implications to cancer therapy.

## 1. Introduction

Cancer remains the second leading cause of death in humans and dogs in the United States, with 1.66 million and 4.2 million diagnoses annually, respectively [1]. This is documented globally as the second-leading cause of death amongst human populations, despite advances in clinical and laboratory research [2]. Systemic therapies remain pivotal in the adjuvant or neoadjuvant setting as a strategy to achieve local tumor control and address micrometastatic spread. New and novel systemic therapies require rigorous safety and efficacy trials before being established as standards of care. Recent evidence also highlights the current problems with the drug development pipeline; both high-costs and high failure rates exceeding 80% in Phase II clinical trials burden patients and clinicians alike, with 51% of failures being related to a lack of treatment efficacy, and 19% due to preclinical safety concerns [3,4,5].

Animal models and tissue cultures yield information about pharmacodynamics and pharmacokinetics and permit monitoring of tumor response to new therapeutics in the laboratory. For decades, drug development trials have used laboratory-based animals with transplanted (i.e., xenograft) and unnaturally developing cancers as preclinical models. Despite a substantial body of research developing and utilizing highly complex mouse models, limitations of evaluating interactions within the tumor microenvironment (TME) remain a significant translational challenge [3]. The study of small animal and laboratory-based models rarely reflects the natural progression of tumors, including intratumor heterogeneity, the tumor microenvironment, and its response to therapy. Other challenges include temporal alterations in biology and the potential interactions that are associated with the host and tumoral immune system or infiltrates [3,4,5]. Tumor organoids, or three-dimensional cellular cultures, have provided an ex vivo framework to study cellular lineage propagation and differentiation. Organoid studies have gained widespread interest as a laboratory model to evaluate treatment response [6]. Despite the known advantages of organoid-based studies, such as higher-throughput experiments and cost-effectiveness compared to xenogenic mouse models, limitations exist and are dependent on cancer cell phenotype. They include low establishment rates, overgrowth of somatic stem cells, and the extended time to develop when used as a co-clinical avatar [4,6]. Additionally, the lack of a TME, including vasculature, stromal cells and immune infiltrates that play a critical role in drug response can limit findings from organoid studies. These TME components are critical in understanding emerging drug strategies, such as immunotherapies.

Immunotherapy, combined with the other pillars of oncology (surgical, radiation, and systemic therapy), has been pivotal in transforming cancer care in the last ten years in medicine. The use of anti-cytotoxic T lymphocyte-associated antigen-4 (CTLA-4) monoclonal antibodies (mAbs) or mAbs targeting the programmed death receptor and its ligand (PD-1 and PD-L1, respectively), has generated tremendous interest in the ability to assess and treat cancer patients by harnessing the immune system, revealing the broad-spectrum and tumor agnostic strategy of such therapies [7,8,9,10,11,12,13,14]. In recent years, tumor-infiltrating lymphocytes (TILs) have become increasingly relevant as therapeutic response markers and prognostic indicators, particularly in breast cancer [15,16]. Interactions between tumor cells, stromal cells, and immune cells (T-regulatory cells [Tregs], natural killer [NK] cells, and tumor-associated macrophages [TAMs]) appear to have significant effects on treatment response and prognosis in patients with cancer [17]. As a result, there has been a shift towards personalized treatments according to individualized tumor characteristics, including genomic profiling, microenvironmental heterogeneity, and tumor-immune interactions.

The dog as a spontaneous model for human neoplasms is considered suitable for bridging the gap between spontaneous diseases and animal models in translational medicine. The dog has a series of advantages compared to murine models, the first of which is genetic homology; the dog shares more than ~650 Mb of ancestral sequences with humans, which are absent in mice [18]. Canine DNA and protein sequences are much more similar to the humans compared to the mouse. Additionally, the ability to evaluate breed relationships may provide information regarding a reduced genetic heterogeneity, considering that the dog has a linkage disequilibrium 100 times higher than humans [18]. Sharing the environment with humans, the availability of care and the survival of dogs even up to an elderly age, make companion animals a good model for environmental exposure to certain exogenous carcinogens as well.

Taken together, clinically relevant models that represent the tumor-host-immune interactions remains important to drive biomarker discovery, therapeutic progression, and treatment success. Companion animal models, in coordination with mouse and other pre-clinical models, may assist in advancing our understanding of naturally occurring cancers, improve drug discovery, and allow for evaluations that are similar to those in human patients over a shorter time interval while benefiting both the animal patient and their family.

## 2. Veterinary Oncology as a Comparative Model

Approximately one in four canine companions and one of every five feline companions will develop naturally-occurring cancer in their lifetime [19,20,21,22]. In the United States, the annual incidence of newly diagnosed canine cancers has reached over four million cases and in Switzerland, the annual incidence rate for dogs at 10 years of age was 718.3 tumors per 100,000 dogs/year [23,24]. The study of cancer in companion animal species has become increasingly relevant in the last decade, generating tremendous interest in comparative oncology. Comparative oncology includes the analysis of naturally occurring cancers in animal species that mirror their human counterparts [24]. The National Cancer Institute/National Institutes of Health (NCI/NIH) developed a comparative oncology platform (Comparative Oncology Trials Consortium) to explore and take advantage of a comparative oncology approach to clinical trials and drug development [24]. The genetics of several canine cancers have been explored in detail and are described elsewhere [25]. A recent study showed that transcriptomic profiles of canine melanoma, osteosarcoma, T- and B-cell lymphoma, and pulmonary carcinoma share similar gene activation patterns to humans and are highly relevant for comparative study [26]. Significant homology in tyrosine kinase and phosphatidylinositol 3-kinase pathways, as well as immune pathways, have all been described [25]. Similarities in pathology and disease behavior have been documented in several tumor types that serve as potential translational models, including canine glioma, osteosarcoma, urothelial carcinoma, melanoma, breast cancers, lymphoma, and lung cancers [5,24,27,28,29]. There are numerous advantages of studying canine and feline models compared to the mouse. In dogs and cats, they are spontaneous tumors that are localized in the same sites as humans and with the same physiological characteristics, develop in immunocompetent subjects and share the same clinical behavior and progression [30]. Moreover, these companions share the same environment and are, therefore, subjected to the same carcinogens. One notable example of the utility of comparative oncology is through the use of non-steroidal anti-inflammatories for muscle-invasive urothelial carcinoma. This was first described in dogs and translated to the human patient population [31,32].

Further studies have significantly highlighted the translational relevance of muscle-invasive urothelial carcinoma (MIUC) between these species, including the evaluation of immune biomarkers and transcriptomic profiles [33,34,35,36,37,38]. Multicentric and non-Hodgkin’s-like lymphomas are among the most prevalent canine cancers and are treated similarly to their human counterparts via a CHOP chemotherapy protocol [24]. A rituximab-like antibody has also been developed in canines [39,40]. Canine osteosarcoma is 20 times more frequent than pediatric osteosarcoma while also sharing many similar biologic features, including primary tumor location, microscopic metastatic disease at diagnosis, altered expression of key proteins such as ezrin, and genetic aberrations of p53, phosphatase and tensin homolog (PTEN), retinoblastoma 1 (RB1), hepatocyte growth factor receptor (MET) and v-erb-b2 erythroblastic leukemia viral oncogene homolog 2 (ERBB2) [18,41]. This, in combination with the shorter lifespan of dogs, makes canine osteosarcoma a highly relevant model for comparative study [41]. The NCI/NIH Comparative Oncology Trials Consortium has recently developed a pipeline for a large-scale standard-of-care control population of canines with osteosarcoma. It has evaluated two new and novel therapies, including rapamycin and a *listeria*-based vaccine vector against HER2/neu [42,43]. Furthermore, we must not forget the studies in canine melanoma, where xenogeneic DNA vaccination against tyrosinase antigen reduced the time of experimentation in humans, given its evidence of use in dogs [30]. More recently, inhaled recombinant IL-15 was also trialed in dogs with naturally occurring metastases from osteosarcoma and melanoma given their comparative relevance [44]. The exact details about the similarities between the underlying genetic mechanisms, tumor development, behavior, and treatment are covered elsewhere and are thus not described in detail here [5,24,45,46,47,48]. Similarly, the utility of advanced and comparative imaging in veterinary patients in a clinical setting with PET-CT, CT, MRI, and others has also been described by others [49,50].

Another tumor that is widely used as a spontaneous model in dogs for humans is hemangiosarcoma. Canine hemangiosarcoma accounts for 5–7% of all canine tumors and Golden Retrievers, German Shepherds, and Boxers are predisposed breeds. It has been shown that in Golden Retriever hemangiosarcoma, there is a significant over-expression of VEGF1 compared to other breeds that instead over-expressed VEGF2. In fact, the blockade of VEGF2 expression in hemangiosarcoma cell lines was associated with a reduction in cell growth in vitro in all breeds except the Golden Retriever, suggesting the importance of evaluating the specific genetic background for that breed [51].

Dogs have similar activated and central T-cell phenotypes that are detectable via flow cytometry, which allows for an accurate assessment of cancer vaccine research. As well, specific major histocompatibility complex (MHC) expression allows for study of cytotoxic T-cell responses to specific neoantigens [52]. One notable difference is that canine neutrophils appear to express cluster of differentiation 4 (CD4), which is not described in humans, although both canines and humans share CD4 expression in lymphocyte populations [53]. Regardless, the repertoire of immune markers and the homology of both myeloid and lymphoid populations between species has been described elsewhere [54,55]. Transcriptomic analysis of canine natural killer (NK) and myeloid-derived suppressor cells (MDSC) has provided further evidence of similarities between species [56,57]. Canine NK cell transcriptional changes appear to be more representative of human than mice in several different environments [57]. Due to these similarities, the investigation of CTLA-4, PD-1, and PD-L1 as specific biomarkers and potential treatment targets in canine and feline cancers has been the focus of ongoing research [58,59,60,61,62,63]. Other studies have targeted OX40, TIM-3, and other co-stimulatory and checkpoint molecules for evaluation in veterinary medicine [55]. In fact, a recent study emphasized the increase of IBA1+ and PD-1+ cells in high-grade compared to low-grade canine mast cell tumors [64].

One of the limitations in veterinary immuno-oncology research has been the lack of caninized antibodies and other immunological reagents [55]. However, this continues to improve as major drug companies and laboratory suppliers see the comparative relevance of this species, with many veterinary schools and comparative research groups working to validate human, mouse, rabbit, or develop caninized reagents for clinical research [55]. Similarly, research groups have continued to develop monoclonal antibodies and checkpoint inhibitors for animals. Most recently, an anti-canine CTLA-4 antibody has been produced and will soon be used in large-scale veterinary clinical trials, closely mimicking their human counterparts [65]. An anti-PDL1 therapeutic is also in development and reported in the veterinary literature [66,67]. As well, adoptive T-cell therapeutics, chimeric antigen receptor T-cell (CAR-T), cancer vaccines, the use of viral immunotherapies, and others have all been evaluated and will continue to be in companion animals with cancer [68,69,70,71,72,73,74,75,76,77,78].

## 3. Tumor-Infiltrating Lymphocytes

**Figure 1:**
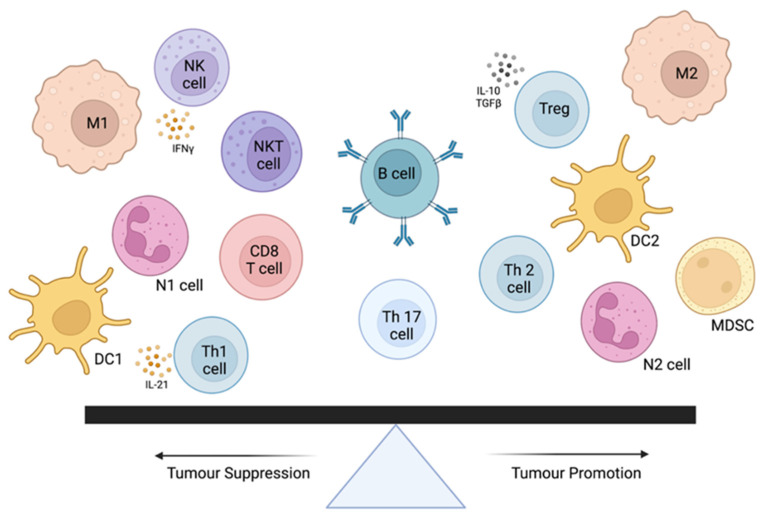
The immune cellular microenvironment of cancer. Pro-tumorigenic immune cell types include T-regulatory cells (Tregs), M2 macrophages, dendritic cells (type 2), myeloid-derived suppressor cells (MDSCs), T-helper 2 (Th2) cells, and neutrophils (N2). Anti-tumor immune cell types include CD8+ T-cells, T-helper 1 (Th1) cells, neutrophils (N1), dendritic cells (type 1), natural killer (NK), and natural killer T-cells (NKT). These cells, their relative abundance, and microenvironmental signals play a critical role in anti- or protumor activity of cancer cells. Salgado et al., Harmonization of the evaluation of tumor infiltrating lymphocytes (TILs) in breast cancer: recommendations by an international TILs-working group 2014 *Ann Oncol* mdu450 first published online 11 September 2014 https://doi.org/10.1093/annonc/mdu450. By permission of Oxford University Press on behalf of the European Society for Medical Oncology.

Tumor-infiltrating lymphocytes are immune cells that are observed within the tumor microenvironment that interact with local tumor cells and tissue stroma. These infiltrates were originally defined in 1989 by Clark et al., followed by Clemente et al. in 1996 [79,80]. Based on the local cytokine milieu, TILs heavily influence local tissue proliferation, angiogenesis, and even metastasis [81]. Their role has been debated in both the human and veterinary literature. However, the recent advancements in immunotherapeutics point to their role as both effector cells (i.e., anti-cancer agent) or as a biomarker for treatment response and prognosis [81]. TILs can transform the TME but can be influenced by local tissue stroma or cancer cells themselves (Figure 1). Alterations in T-regulatory cells (Tregs) have vast implications for converting immunologically cold to hot tumors that are more responsive to immunotherapy [82]. Therefore, our careful manipulation of these local tissues and immune-tumor-cell interactions has vast implications for improving response and prognosis in our human and animal patients with cancer.

### 3.1. TILs in Human Oncology

Among several cancer types, TILs have been well studied in breast carcinomas (BC) but has been associated with outcomes in several tumor types [83]. The utility of TILs to predict response to neoadjuvant chemotherapy (NACT) in women with breast cancer has been evaluated, proving that patients with increased TIL density have a higher incidence of pathologic complete response (pCR) [15,84,85,86,87]. TIL infiltration is also considered a negative prognostic factor among several histologic breast cancer subtypes, namely the luminal breast cancer subtype. TIL interactions with cancer cells or the stroma are independent prognostic factors in patients with breast, colorectal, and ovarian cancers [87,88,89,90,91]. Cerebelli et al. developed a tumor immune profile (TIP) to predict response to NACT in triple-negative breast cancers [92]. Patients that were documented as TIP-positive were more likely to achieve pCR. TILs have also been utilized for risk stratification for breast cancer patients to receive adjuvant therapies. For example, Ahn et al. found that tumors with high TIL density (≥60%) showed a higher risk for recurrence compared to those with intermediate and low density, but only in luminal breast cancer [93]. In general, high TILs are prognostic in the Triple-negative and HER2+ breast cancer subtypes and predict response to immunotherapy [94]. TIL assessment may translate from bench to bedside, guiding clinical decision-making for both the physician and patient.

TILs are also important biomarkers in cutaneous melanoma. Clark et al. in 1989 determined that 8-year survival rates were lower (59%) in patients with absent TILs compared to those with non-brisk and brisk TIL patterns (75% and 88%, respectively) [79]. This was later inversely associated with sentinel lymph node metastasis, a known significant prognostic factor in cutaneous melanoma [95]. TIL infiltration was also independently associated with disease-specific survival [96].

Response to immunotherapy appears to be affected by TIL infiltration in both breast cancer and cutaneous melanoma. In the KEYNOTE-086 trial, evaluating the efficacy of pembrolizumab, breast cancer patients with high TILs had an improved clinical response to anti-PD1 therapy [97]. In cutaneous melanoma, TIL infiltration appears to be inconsistently related to treatment response [83]. However, TIL infiltration in lymph node metastatic lesions is higher in ipilimumab responders than non-responders [98]. CD8+ T-cell density at the tumor margin also appears to predict response to pembrolizumab in cutaneous melanoma patients [99].

Our understanding of the TIL-tumor microenvironment interaction has become so vast and complex that tertiary lymphoid structures (TLS) have been described as a secondary lymphoid expansion of TILs within the microenvironment or stroma. Similar to a lymph node, these TLSs can have germinal centers and an extensive microenvironment of their own [100]. These concepts will continue to be important for recognizing spatial influences on tumor behavior, including finding biomarker signatures that are associated with recurrence and prognosis [100,101,102].

### 3.2. TILs in Veterinary Oncology

Limited studies have evaluated TILs in veterinary oncology with some historical emphasis on mammary carcinomas in dogs and cats, cutaneous histiocytoma in dogs, and seminoma in dogs. However, there has been increasing interest in the last decade given the implications that are documented in humans. Several groups have attempted to assess the prognostic significance of immune infiltrates and host-immune interactions at the histologic level in many immunogenic tumor types (Table 1). The relevant studies are summarized below.

#### 3.2.1. Transmissible Venereal Tumor (TVT)

**Figure 2:**
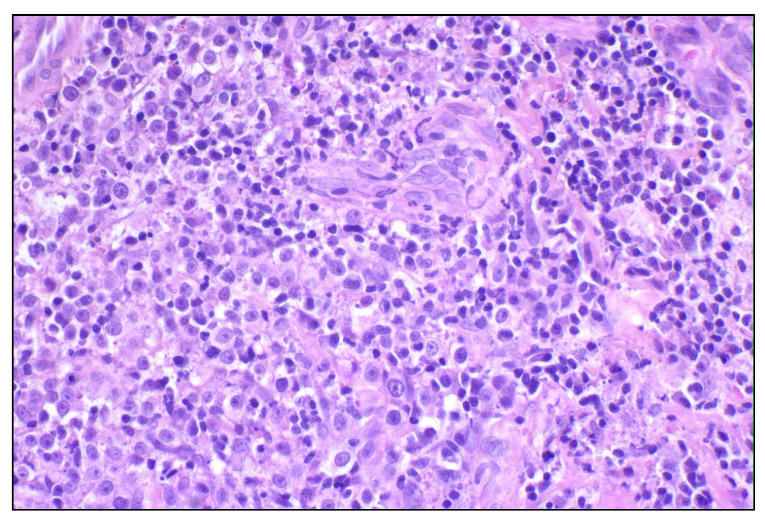
Canine transmissible venereal tumor, vagina, hematoxylin and eosin 40×: sheet of neoplastic round cells, characterized by a moderate amount of lightly eosinophilic cytoplasm, intermediate nucleus-cytoplasmic ratio, and a round nucleus with prominent nucleolus. Intermingled with neoplastic cells are numerous tumor infiltrating lymphocytes.

Canine TVT is a round cell tumor that is suggested to be of the histiocytic lineage that is transmitted within the canine species [105] (Figure 2). The tumor commonly presents as nodular growths along mucocutaneous junctions (oral cavity, penis/prepuce, vestibule, vagina) and has a low rate of metastasis. Generally, TVT has a predictable growth pattern: the initial progressive phase (P-phase), where the tumor may continue to grow for up to 3 to 6 months, followed by a stationary phase (S-phase) where tumor growth is halted for months to years, and last, a regression phase in younger animals (R-phase) where complete eradication of the tumor is achieved. The R-phase is typically mediated by a distinct increase in TIL infiltration histologically [103,104]. Infiltration of CD8+ TILs is correlated with interleukin-6 (IL-6) production, restoration of natural killer cell activity, and apoptosis of TVT cells [103,105]. Transmissible venereal tumors were among the first documented observations of TILs activity in veterinary medicine and provides a viable model for tumor immunogenicity. As well, it should be noted that this was a pivotal time where TILs were first used as an immune biomarker in veterinary oncology; in particular, early-regressing tumors had a significantly higher CD8+ TIL infiltration than late-regressing TVTs [104].

#### 3.2.2. Canine and Feline Mammary Carcinoma

Canine and feline mammary carcinomas are well recognized as models of human breast cancer [24]. Similar to their human counterparts, hormone receptor expression and hormonal influence play a role in developing benign and malignant breast cancers. Similar subtypes (luminal A, luminal B, HER2-overexpressing, and triple-negative) have been described, closely mimicking human breast cancer [24,63,131,132,133]. Mutations in BRCA1 and 2 and higher expression profiles of the gene have also been described in canine patients with mammary cancer [106,134]. Systemic therapies are pursued dependent on tumor histologic subtype and based on tumor size, grade, or nodal involvement, similar to humans [135,136,137]. Initial pharmacokinetic profiles of iniparib were evaluated in dogs and translated for use in humans, given similarities in cross-species metabolism [138].

**Figure 3:**
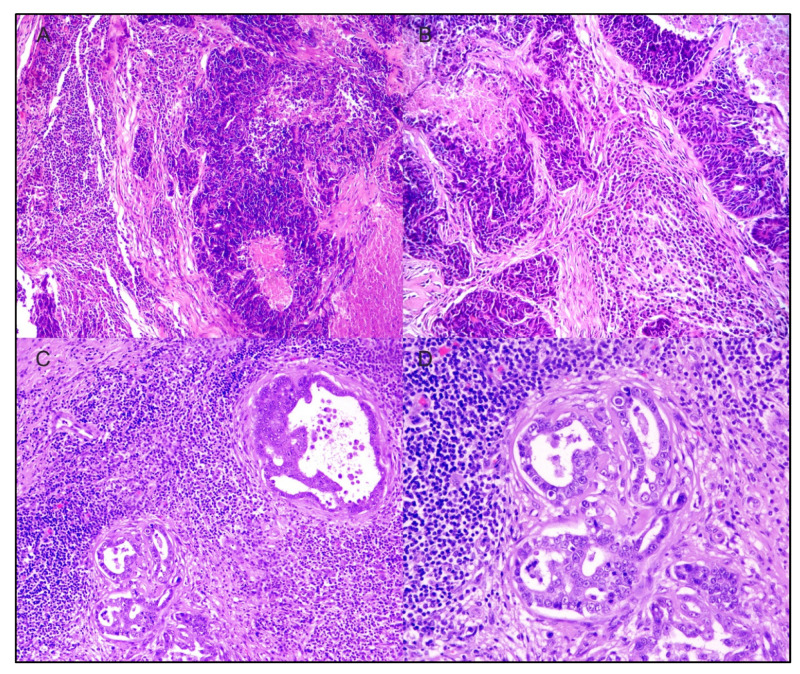
(**A**,**B**) Canine mammary comedocarcinoma histological grade III, hematoxylin and eosin. (**A**) The tumoral stromal area is massively effaced by tumor infiltrating lymphocytes (TILs), 10×; (**B**) higher power magnification showing mainly lymphocytes and plasma cells among TILs, 20×. (**C**,**D**) Feline mammary simple tubular carcinoma, histological grade II, hematoxylin and eosin. The stromal area is strongly infiltrated by TILs, 10×; higher magnification depicting mainly small lymphocytes among TILs.

**Figure 4:**
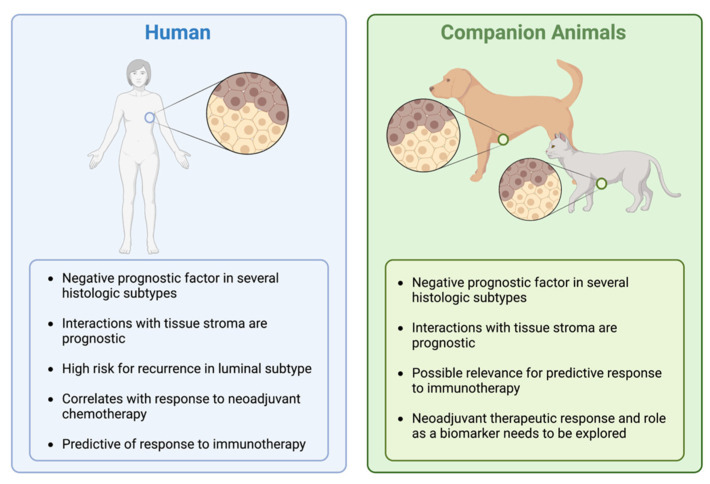
Comparative similarities of TIL evaluation in human and companion animal breast cancer. Several similarities exist between species, including immune checkpoint expression of TILs; however, further evidence is required in companion animals to elucidate the role of TILs as a biomarker for response to immunotherapy. Neoadjuvant chemotherapy and TILs as a marker for therapeutic response also require further study in companion animals.

In early studies evaluating TILs in mammary carcinoma, TILs were abundant in malignant tumors compared to benign and normal mammary tissue [106] (Figure 3). A significantly greater number of TILs were also noted in metastatic lesions than primary malignant tumors. In a follow-up study by the same group, TIL infiltration was evaluated in 47 cases of canine mammary carcinoma and scored based on TIL distribution and intensity [107]. Higher TIL scores were significantly associated with histologic grade and lymphatic invasion though the association with molecular phenotype (luminal A, luminal B, basal, HER-2-overexpressing) was not significant. On subgroup analysis, a significantly higher infiltration of CD3+ T-lymphocytes (mean positive area/1.6 mm^2^) was associated with high histologic grade (0.118 ± 0.119, *p* = 0.035) and presence of lymphatic invasion (0.118 ± 0.112, *p* = 0.008) [107]. Immune checkpoints such as CTLA-4 and PD-1/PD-L1 and TIL infiltration have also been evaluated in felines. Serum PD-1 and PD-L1 levels are significantly higher in cats with HER2-positive and triple-negative mammary carcinomas, similar to those that are documented in humans [63]. PD-L1 expression is also considerably higher in feline HER2-positive mammary carcinomas. Total PD-1 and PD-L1 scoring of TILs and cancer cells are significantly elevated in HER2-positive mammary carcinomas in felines compared to the triple-negative normal-like subtype, similar to what is seen in human HER2-positive breast cancer [63]. V-domain immunoglobulin suppressor of T-cell activation (VISTA)-positive TILs have also been evaluated in feline mammary carcinoma, and by stratifying based on subtype, cats with HER2-positive disease had a significantly higher proportion of VISTA-positive TILs than those with the triple-negative subtype (*p* = 0.0138) [108]. However, this was not correlated with outcome. In this same study, the percentage of VISTA-positive TILs was also higher in grade II (*p* = 0.0025) tumors when compared to grade I [108]. Cytokines that were associated with tumor growth and metastasis (IL-1 and IL-6) are upregulated, particularly in canine metastatic mammary carcinoma samples. These same cytokines remain elevated in malignant mammary tumors compared to benign and normal mammary tissue [106]. The CD4+/CD8+ ratios of TILs are also significantly increased in metastatic groups, and those canine patients with higher CD4+ TILs have a poorer overall survival based on log-rank analysis [109]. CD4+ TIL infiltration was seen in a higher proportion of those dogs with lymph node metastasis. Higher CD8+ TILs in this same study correlated with increased survival based on log-rank analysis [109]. These prognostic implications were further validated when evaluating adnexal CD3+ TILs in canine mammary carcinomas [110]. Higher numbers of CD3+ TILs within the adnexal mammary gland were significantly correlated with poorer survival, with 50% alive at 18 months (TIL count > 107) compared to 84% (TIL count ≤ 107). This was also significantly associated with higher histologic grade, lymph node status, and distant metastasis. Similarly, higher intra-tumoral CD3+ TILs were correlated with poorer overall survival, although this approached significance [110]. Approximately 41% of patients were alive at 18 months (TIL count > 256) compared to 72% (TIL count ≤ 256). These TILs were also significantly correlated with more invasive and biologically aggressive histologic subtypes [110]. The infiltration of these TILs, specifically within the adnexa, is thought to be mediated by chemokine ligand-2 (CCL2) and CCL5 that are produced by tumor cells and other tumoral leukocytes [139]. This is well-documented in human breast cancers, providing a comparative landscape for canine mammary carcinoma (Figure 4). Given the increasing interest in comparative TIL assessment, Muscatello et al. recently proposed a standardized approach to evaluate TILs in canine mammary carcinomas, using the internationally standardized approach in human breast cancer that was developed by Salgado and colleagues [16,111]. Interestingly, contrary to human breast cancer, high stromal TIL infiltration was correlated with positive lymph node metastasis but was proposed as a Type I error due to small sample size [111].

#### 3.2.3. Canine Oral Malignant Melanoma (OMM)

Canine OMM has proven translational relevance to human mucosal and cutaneous melanoma and provides an accurate model to study the disease in a naturally occurring animal model [30,140]. The genomic landscape and similarities of human mucosal and canine oral melanoma have been well-defined recently [141,142,143]. Canine OMM has also matured into a highly relevant translational model for immunotherapeutics given its similar immune features and repertoire for checkpoint inhibitors [30,59,66,67,144]. Given its immunogenicity, a therapeutic xenogeneic human tyrosinase bacterial DNA plasmid vaccine has been licensed dogs with OMM [145].

TILs, as well as circulating T-cells, have been evaluated in canine OMM [112]. Flow cytometric evaluation of peripheral blood in canine OMM patients revealed significant increases in TILs compared to healthy controls [112]. Similarly, percent Tregs within tumor tissue were significantly increased (2.5-fold increase in the total number of Tregs when compared to peripheral blood) [112]. More recently, the histologic evaluation of TILs in canine melanocytic neoplasms was investigated by Porcellato et al. [113]. In melanocytic tumors with high CD20+ TIL infiltration, patients were at higher risk of death related to tumor progression, metastasis, or recurrence. The 2-year survival with low CD20+ TIL infiltration was 79%, whereas, with high CD20+ TIL infiltration, this probability decreased to 24%. In canines, this was one of the first studies to suggest the use of TIL infiltration as a biomarker in canine melanoma [113]. Interestingly, in this same study, significant associations were found between TILs and pathologic features such as mitotic count, melanin-pigmentation, and cellular pleomorphism [113]. In 2015, an NIH-supported study evaluated IL-12 therapy in dogs with OMM [75]. After treatment, T-cell populations and intra-tumoral CD8+ T-cell infiltration increased with treatment, indicating a potential biomarker for response to cytokine therapy [75]. Yasumaru and colleagues evaluated TIL patterns in 50 samples of canine OMM [114]. Patients with higher survival rates had higher TIL scores and an increased frequency of CD8+ TILs [114]. More recently, Stevenson et al. evaluated PD-1, PD-L1, and PD-L2 gene expression as well as TILs in canine melanoma [115]. A significantly higher relative abundance of CD3+ TILs (38 ± 11.5%, mean ± SEM; *p* = 0.026) were observed in groups with the highest PD-1 expression when compared to low expressors. Although there is a small number of studies compared to human trials, canine melanoma proves to be comparatively relevant. Further work is needed to clarify the role of TILs as biomarkers for response to therapy and prognostication in canine OMM.

#### 3.2.4. Canine Osteosarcoma (OSA)

Canine OSA has known clinical, pathologic, and molecular relevance to OSA in humans [24,41,45,146,147,148]. Canine OSA occurs more commonly in the canine population than in children, making it an excellent model for studying novel therapies. Immune infiltrates are documented in metastatic lesions and remain an attractive target for treatment [116,117]. Regan and colleagues have studied the effects of specific drug combinations to enhance the efficacy of therapy in the metastatic disease setting [118]. Immunosuppressive macrophages (M2 macrophages) support angiogenesis, facilitate extravascular escape leading to metastasis and immune escape [149]. Regan’s group showed that using a combination of losartan (angiotensin II receptor blocker) and toceranib phosphate (tyrosine kinase inhibitor), the tumor immune suppression that was imposed by M2 macrophages could be reversed and led to improvements in progression-free survival (111 days compared to 57 days) in the gross pulmonary metastatic disease setting [118]. Importantly, this comparative work has now led to a clinical trial utilizing losartan in pediatric OSA (clinicaltrials.gov, identifier: NCT03900793). Similarly, a HER2/Neu *listeria*-based vaccine vector has been used in dogs with osteosarcoma [43]. This has now developed into a large-scale clinical trial with the NIH (COTC026), given its potential to prolong survival in canine patients with osteosarcoma and could be translated to initiate human trials. Interestingly, T-cell infiltrates within metastatic lesions were documented after vaccination with a HER2/Neu *listeria*-based vaccine [43]. From a prognostic role, decreased ratios of peripheral blood and lymph node CD8+:Tregs are associated with poorer survival [119]. This closely mimics what has been seen in pediatric OSA patients [150]. In more recent work, Cascio et al. described immune checkpoint expression (PD-L1, CD270, and B7H3) in canine osteosarcoma and their relationship with peri- or intra-tumoral TILs [120]. A high expression of these immune checkpoints on tumor cells resulted in log-fold increases of peritumoral T-cell infiltration without intra-tumoral penetration. Despite much of the work being performed in canine osteosarcoma, the absolute prognostic role of TILs remains to be elucidated in canines with osteosarcoma.

#### 3.2.5. Canine Histiocytic Neoplasms

Histiocytomas are benign cutaneous neoplasms of dogs, mainly located on the head, ears, neck, and more frequently occurring in younger animals. Histiocytomas occur in all breeds but purebred dogs are predisposed, particularly Boxers and Dachshunds [121]. Histiocytic sarcoma (HS), the malignant variant, is considered rare in humans. However, in dogs, the disease is more common than in humans and particular breed overrepresentations have been documented in Bernese Mountain Dogs, Rottweilers, Flat-coated Retrievers, and Miniature Schnauzers [151,152,153,154,155,156]. Several genetic alterations have been documented that show commonality between humans and dogs including the loss of PTEN and mutations in KRAS [154,157].

**Figure 5:**
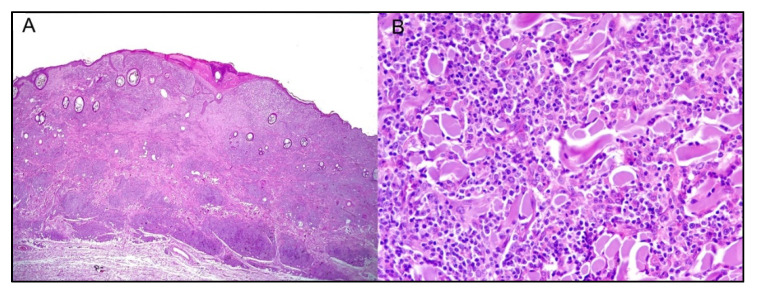
Canine cutaneous histiocytoma, hematoxylin, and eosin: (**A**) the dermis is expanded by a dome-shaped densely cellular neoplasm, characterized at the base by a densely basophilic area suggestive of lymphocyte infiltration. (**B**) The neoplasm is composed of round histiocytic cells to which numerous lymphocytes are mixed, that are responsible for tumor regression, 20×.

Histiocytomas are tumors of Langerhans cells, grossly visible as usually single dome-shaped erythematous nodules, which may undergo spontaneous regression and this process is mediated by the infiltration of lymphocytes at the base of the neoplasm (Figure 5). These lymphocytes mediate the lysis of neoplastic histiocytes, since it has been shown that the tumor-infiltrating lymphocytes are highly enriched with CD8+ T-cells, capable of mediating regression [121,122,123,158]. In histiocytic sarcoma of Flat-Coated Retrievers, TIL infiltration had been described but not correlated with outcome [159]. A recent study by Lenz and colleagues suggests that increased CD3+ and granzyme B+ TIL infiltration are associated with more favorable outcomes in dogs with histiocytic sarcomas [124]. Patients with high CD3+ TIL density (above the median cutoff) in this study had an improved median survival time when compared to those with low CD3+ TIL density (400 days vs. 150 days, *p* = 0.029). This held true for granzyme B+ TIL density (*p* = 0.035) and approached significance for FOXP3+ TIL density (*p* = 0.11) [124]. Additionally, transcriptional analysis showed that increased T-cell transcripts were associated with improved survival in canine pulmonary histiocytic sarcoma, specifically.

#### 3.2.6. Canine Gliomas

Canine gliomas are considered a good naturally occurring model for the human disease and represent around 40–70% of primary canine brain tumors [125,160].

TIL populations in canine glioma were recently characterized by Pi Castro and colleagues [125]. The phenotype of TILs was predominantly composed of CD3+ T lymphocytes and a high number of CD3+ TILs were associated with high grade glioma, distributed with a specific spatial distribution within the tumor stroma and in the brain-tumor junction. In contrast, low-grade gliomas were infiltrated by a low number of CD3+ TILs that were scattered in the tumor stroma. FOXP3+ lymphocytes shared a similar distribution of CD3+ TILs. A similar study evaluating immune cell infiltration in canine gliomas found that high-grade tumors had a significantly higher number of FOXP3+ cells/high-powered field when compared to low grade (*p* = 0.006) [126]. Similarly, Mac387+ and CD163+ cells were significantly higher in high-grade gliomas when compared to low (*p* = 0.01, *p* = 0.01, respectively). Overall, TILs in canine glioma display similar immunophenotypic features to humans, supporting the dog as a good spontaneous model [125].

#### 3.2.7. Canine Urothelial and Prostate Cancer

**Figure 6:**
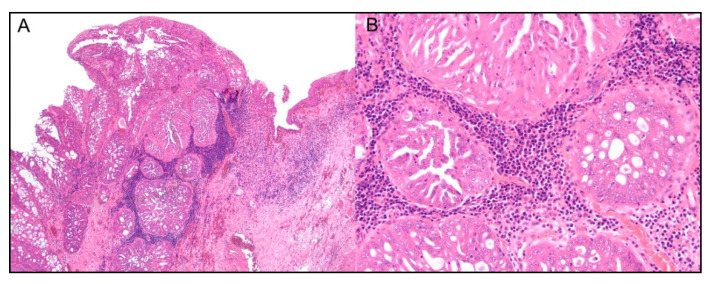
Canine urothelial cell carcinoma, hematoxylin, and eosin, urothelial cell carcinoma. (**A**) Moderate numbers of lymphocytes are multifocally present within the fibrovascular stroma adjacent to the neoplastic proliferative front, 4×. (**B**) Focally extensive increase in numbers of plasma cells (tumor infiltrating plasma cells, TIPs) surround cancer nests, 20×.

Urothelial carcinomas (UC) are the most common genitourinary tumors in dogs and closely mimic the muscle-invasive phenotype that is seen in humans [33,36,161] (Figure 6). The BRAF V595E mutation is seen in roughly 85% of canine patients with UC, which has prompted recent investigation into the efficacy of vemurafenib in dogs [162,163,164,165,166]. Several groups have documented the immune landscape and mRNA expression of canine UC, revealing its utility as a relevant translational model [37,38,167,168,169]. A recent study revealed increased numbers CD3+ T-cells (though not statistically significant) in TME-Hot tumors when compared to TME-Cold [38]. Tumors with higher CD3+ T-cell staining also showed enriched gene expression that was associated with immune-related hallmarks (such as T-cell receptor signaling pathways). However, specific TIL evaluation as a biomarker is still lacking in canine UC.

In human prostate cancer, inflammation influences tumor progression with immune infiltrates being the main drivers of that effect [170]. For canine prostate cancer, recent evidence shows that T and B lymphocytes are increased in the tissue and that intra-tumor CD3+ TILs and granzyme B+ cell estimation are correlated with survival [127,129]. Also, it has been shown that increased Tregs are associated with poor prognosis showing parallels with what is observed in human tumors [128,170].

#### 3.2.8. Canine Pulmonary Carcinoma

**Figure 7:**
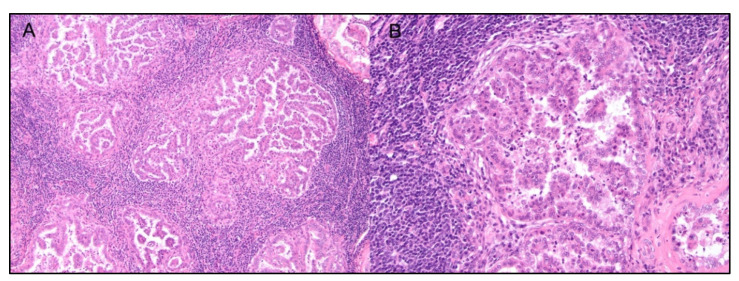
Canine pulmonary carcinoma (with metastasis to right bronchial lymph node) classified as grade III, hematoxylin, and eosin. (**A**) High-grade TILs: numerous lymphocytes are distributed adjacent to the cancer nests, 10×. (**B**) Higher magnification, 20×.

Pulmonary carcinoma is the most common pulmonary neoplasm in both dogs and humans with comparative relevance given similarities in their molecular underpinnings [171] (Figure 7). Canine pulmonary carcinomas are thought to be of bronchioloalveolar origin corresponding to approximately 85% of the total primary lung tumors; the rest include adenocarcinomas, adenosquamous, and squamous cell tumors [172].

Regarding the immunological profile of dogs with pulmonary carcinoma, intra-tumoral, and peritumoral FOXP3+ expression has been assessed, demonstrating low numbers of intra-tumoral Tregs correlate with an improved survival (*p* = 0.0074) when compared with high [130]. Another study investigated the inflammatory milieu of neutrophilic leukocytosis in two cases of canine pulmonary carcinoma, a significant increase in IL-6 and G-CSF production was identified when compared to normal lung control tissue. IL-6 is produced by a multitude of inflammatory cells and its role in tumor metastasis and as a prognostic marker in human non-small cell lung cancer (NSCLC) has been previously established [173,174,175]. Similarly, a previous study identified the expression of G-CSF in human NSCLC as a prognostic biomarker [176].

These findings in dogs warrant further investigation in the context of the immunological microenvironment and association of the expression of certain cytokines and prognosis.

## 4. Comparative TIL Assessment and Future Directions

The comparative assessment of TILs in companion animals represents challenges but also vast opportunities in oncology. Spatial relationships within the tumor microenvironment should be explored, and direct assessment of TILs remains pivotal as biomarkers for predictive and prognostic modelling [83]. Comparative oncology platforms present a unique opportunity whereby investigators can reciprocally evaluate TIL biomarkers and other relevant metrics that may be translated to clinical decision-making for both species. New and early-stage therapeutics directed at the immune TME can be studied in dogs and cats, given their similarities in metabolism and ability to attain relevant pharmacokinetic and pharmacodynamic, as well as safety/efficacy data [24]. For those that are interested in evaluating treatment response to immunotherapeutics, veterinary patients provide a naturally occurring and highly relevant translational model despite the requirement for animal-specific monoclonal antibodies. Biomarkers, such as CD8+ TIL density, have already been associated with improved survival and a lower rate of metastasis in humans and dogs [177]. Similarly, in both species, the infiltration of CD4+ T-cells that impose immunosuppressive behavior within the TME is associated with poorer overall survival and risk of lymph node metastasis [177]. CD3+ T-cell infiltration may also impact angiogenesis, both documented in human and canine breast cancers [81]. As well, the specific utilization of TILs for vaccine or adoptive cell therapy development are largely unexplored in companion animal models and warrants further investigation. Other important spatial relationships may be discovered through comparative assessment at a larger scale and across several tumor types.

Comparative assessment across species can be boosted by computational tools, such as machine learning and deep neural networks, as has been shown in canine and mouse models [178,179,180]. For example, after automated single-cell identification using image analysis, TIL scoring that considers the abundance and spatial location of TILs can be performed using methods that are similar to those that have been developed for computational human histopathology [181,182,183]. This will enable reproducible evaluation of prognostic and predictive value of these metrics in veterinary patients in translational studies and clinical trials. Spatially-resolved immune infiltration analysis has unveiled intriguing cancer biology and clinical relevance for human cancers, and we anticipate similar advances for the studies and treatment of animal cancers. Ultimately, the interchange of tools and common knowledge can lead to advances in medicine for both human and non-human species [184].

## 5. Conclusions

Comparative immuno-oncology and TIL assessment represent a strong naturally-occurring clinical model that can be used to benefit biomarker and treatment discovery for human and non-human clinical practice. Information from one species may translate to another and appropriate naturally-occurring model systems, such as companion animals, may expedite the translational drug pipeline. Some fundamental differences also exist between the incidence of certain human and animal cancers across species and, therefore, comparative oncology also represents a pivotal opportunity to study mechanisms, biomarkers, and treatments for rare cancers that may appear more frequently in a non-human species.

It should be noted however, standardized practices are lacking between current veterinary studies for TIL-assessment. Therefore, a standard guideline of TIL-assessment for veterinary oncologists and pathologists, may only improve comparative relevance. There remains a collaborative need between human and veterinary oncologists as well as cancer researchers to work alongside one another to advance discovery.

## Figures and Tables

**Table 1:** Summary of recent findings of TIL studies in veterinary oncology.

Tumor Type	Summary of Findings	Reference (s)
Canine Transmissible Venereal Tumor	TIL infiltration correlates with regression of the primary lesion	[103,104]
	Infiltration of CD8+ TILs correlates with apoptotic behavior	[103,105]
Canine & Feline Mammary Carcinoma	TIL infiltration is high in canine malignant mammary carcinoma	[106,107]
	Metastatic lesions in canine mammary carcinoma have higher TIL infiltrates	[106]
	PD-1 and PD-L1 scores of TILs are elevated in HER2+ feline mammary carcinomas	[63]
	VISTA-positive TILs are significantly higher in feline HER2+ mammary carcinoma compared to triple-negative	[108]
	Lower CD4+:CD8+ TIL ratio in metastatic sites in canines	[109]
	High CD4+ TIL infiltration correlates with a poor prognosis in canines	[109]
	High CD3+ TIL within adnexa correlates with more aggressive biologic and histologic features and poorer survival	[110]
	A standardized approach for TIL assessment has been validated in canine mammary carcinoma and reflects the methods proposed by the International TILs Working Group	[111]
Canine Oral Melanoma	Canine melanoma has high TIL infiltration	[112,113]
	CD20+ TILs are associated with tumor progression, metastasis, and recurrence	[113]
	CD20+ TILs are associated with poorer overall survival	[113]
	TIL infiltration is associated with pathologic features and malignancy	[113]
	Higher TIL scores and an increased CD8+ TIL infiltration is associated with increased survival	[114]
	High tumoral PD1 expression correlates with increased CD3+ TIL infiltration	[115]
Canine Osteosarcoma	TIL infiltration is documented in metastatic lesions	[43,116,117]
	Therapeutic manipulation of tumor-suppressive macrophages led to improvement in survival in metastatic osteosarcoma	[118]
	Decreased peripheral blood and nodal CD8+:Treg ratios are associated with poorer survival	[119]
	Higher immune checkpoint expression results in decreased intra-tumoral penetration of TILs	[120]
Canine Histiocytic Neoplasms	CD8+ TILs contribute to regression of benign histiocytomas	[121,122,123]
	CD3+ T cell and granzyme B+ TIL infiltration is associated with improved outcomes in histiocytic sarcoma	[124]
Canine Glioma	High CD3+ TIL infiltration is associated with high-grade tumors	[125]
	FOXP3+ TILs share a similar distribution to CD3+ TILs and association with grade	[125,126]
Canine Urothelial & Prostate Cancer	T and B lymphocytes are increased in canine prostate cancer	[127]
	CD3+ lymphocytes are positively correlated with survival	[127]
	Tumor-infiltrating Tregs are associated with a poor prognosis	[128]
	Granzyme B+ TILs are positively associated with survival	[129]
Canine Pulmonary Carcinoma	Low numbers of intra-tumoral Tregs correlate with improved survival	[130]
